# Caspase-3 expression in tumorigenesis and prognosis of buccal mucosa squamous cell carcinoma

**DOI:** 10.18632/oncotarget.20494

**Published:** 2017-08-24

**Authors:** Jer-Shyung Huang, Cheng-Mei Yang, Jyh-Seng Wang, Huei-Han Liou, I-Chien Hsieh, Guan-Cheng Li, Sin-Jhih Huang, Chih-Wen Shu, Ting-Ying Fu, Yun-Chung Lin, Luo-Ping Ger, Pei-Feng Liu

**Affiliations:** ^1^ Department of Radiology, Kaohsiung Veterans General Hospital, Kaohsiung, Taiwan; ^2^ National Yang-Ming University School of Medicine, Taipei, Taiwan; ^3^ Department of Stomatology, Kaohsiung Veterans General Hospital, Kaohsiung, Taiwan; ^4^ Department of Dental Technology, Shu-Zen Junior College of Medicine and Management, Kaohsiung, Taiwan; ^5^ Department of Pathology and Laboratory Medicine, Kaohsiung Veterans General Hospital, Kaohsiung, Taiwan; ^6^ Department of Medical Education and Research, Kaohsiung Veterans General Hospital, Kaohsiung, Taiwan; ^7^ Department of Pathology, China Medical University Hospital, Taichung, Taiwan; ^8^ Institute of Biomedical Sciences, National Sun Yat-Sen University, Kaohsiung, Taiwan; ^9^ Department of Optometry, Shu-Zen Junior College of Medicine and Management, Kaohsiung, Taiwan

**Keywords:** buccal mucosa squamous cell carcinoma, caspase-3, cleaved caspase-3, tumorigenesis, prognosis

## Abstract

Buccal mucosa squamous cell carcinoma (BMSCC) is the most common oral cancer in Southeast Asia. Caspase-3, a key molecule in regulating apoptosis, promotes the malignancy of various cancers. However, its role in BMSCC is unknown. Herein, we evaluated the association of caspase-3 expression with tumorigenesis and prognosis in BMSCC patients. Immunohistochemical staining indicated that the expression levels of cleaved caspase-3 (*p*<0.001) and caspase-3 (*p*<0.001) in 185 BMSCC tissues were significantly higher compared to those in the tumor adjacent normal tissues. Moreover, the high expression of caspase-3 was associated with poor pathological outcomes [advanced pathological stage (*p*=0.029) and larger tumor size (*p*=0.002)] and poor disease-free survival in patients receiving postoperative radiotherapy (*p*=0.030). Moreover, the low co-expression of cleaved caspase-3 and caspase-3 was associated with better disease-specific survival in patients with early pathological stage (I + II, *p*=0.018) or without lymph node invasion (*p*=0.043) compared to the positive/high expression of either or both cleaved caspase-3 and caspase-3. Taken together, cleaved caspase-3 and caspase-3 could be biomarkers for tumorigenesis in BMSCC patients. Cleaved caspase-3 and/or caspase-3 might be prognostic biomarkers for certain stages of BMSCC.

## INTRODUCTION

Oral squamous cell carcinoma (OSCC), the sixth most common malignant tumor in the world and the fourth leading cause of cancer-related death among males in Taiwan, occurs in the oral cavity, including the tongue, buccal mucosa, and lip [1]. In contrast to other sites of oral cancer, the incidence of buccal mucosa squamous cell carcinoma (BMSCC) is higher in Southeast Asia, including Taiwan, due to the high rates of exposure to betel quid often mixed with tobacco [2]. BMSCC also accounts for approximately 10% of all oral cancer cases in North America and Western Europe [3]. BMSCC patients have poor prognosis with a locoregional recurrence rate of up to 57% and 5-year survival rate of approximately 50% [4]. Thus, there is an urgent need to identify novel therapeutic targets for BMSCC.

Apoptosis, also known as programmed cell death, is a process with typical morphological characteristics including reduction in cell size, condensation of the cytoplasm, blebbing of the plasma membrane, and fragmentation of chromatin and DNA into oligonucleosomes [5]. Its main functions in organisms are to physiologically remove abnormal cells during development and maintain tissue homeostasis [6]. A dysfunctional apoptotic system can lead to either excessive removal of normal cells or prolonged survival of abnormal cells. Therefore, dysregulation of apoptosis is involved in the pathogenesis of a variety of diseases such as cancer [7], neurological disorders, cardiovascular disorders, and autoimmune diseases [8].

Caspases give rise to active signaling molecules that participate in apoptosis and are classified by their mechanisms of action, which include initiator caspases (caspase-8 and caspase-9) and executioner caspases (caspase-3, caspase-6, and caspase-7) [9]. Caspase-3, a major effector caspase in apoptotic pathways, is an inactive 32-kDa proenzyme (caspase-3). It is cleaved at an aspartate residue to yield a p12 and p17 subunit to form the active caspase-3 enzyme (cleaved caspase-3) [10] that is responsible for morphological and biochemical changes in apoptosis [11] and is useful in scoring the apoptotic index. Aberrant caspase-3 protein expression has been extensively studied in many cancers, such as gastric cancer [12], hepatocellular carcinoma [13], prostate carcinoma [14], glioblastoma [15], melanoma [16], breast carcinoma [17], pancreatic ductal carcinoma [18], non-small cell lung carcinoma [19], and oral SCC [20–22]. However, its expression and clinical role in BMSCC have not been reported. In the present study, we aimed to investigate the expressions and roles of both cleaved caspase-3 and caspase-3 in tumorigenesis and prognosis of BMCC by using tissue microarray (TMA) consisting of a series of 185 surgically resected BMSCC tissues.

## RESULTS

### The demographical characteristics and pathological outcomes and their impact on the survival of patients with BMSCC

A total of 185 patients with BMSCC were eligible for this study as listed in Supplementary Table 1. The female patients were fewer in number (n=4, 2.2%), and 181 (97.8%) patients were male; the average age of all patients was 52.43 yrs (range: 25–85 yrs). Fifty-one (27.6%), 125 (67.6%), and 9 (4.9%) patients had well, moderate, and poor cell differentiation states, respectively. Sixty-five (35.1%), 47 (25.4%), 19 (10.3%), and 54 (29.2%) patients had tumors of pathological stages I, II, III, and IV, respectively. Seventy (37.8%), 70 (37.8%), 12 (6.5%), and 33 (17.8%) patients had T classification of T1, T2, T3, and T4, respectively. In total, 141 (76.2%) patients were devoid of lymph node invasion. Nineteen (10.3%) and 25 (13.3%) patients had N1 and N2 lymph node metastases, respectively. In addition, 51 (27.6%) and 4 (2.2%) patients had received postoperative radiotherapy (RT) and postoperative chemotherapy (CT), respectively. With a median follow-up of 72.63 (range, 2.87–232.97) months, the overall survival rate was 57.74 ± 3.64% at 5 years, 38.67 ± 4.03% at 10 years and 23.76 ± 5.23% at 15 years. The disease-specific survival (DSS) rate was 60.51 ± 3.63% at 5 years, 50.73 ± 4.18% at 10 years and 41.65 ± 5.47% at 15 years. The disease-free survival (DFS) rate remained constant at 54.63±3.72% after 3 years had passed from the time of initial resection. In addition, the median overall survival time was 7.03 ± 1.08 years, and the median DSS time was 10.56 ± 1.81 years. The overall survival, DSS and DFS curves are shown in Supplementary Figure 1.

The impacts of various pathological outcomes in survival of BMSCC patients were evaluated by univariate analysis. Our data showed that BMSCC patients with moderate (*p*=0.045) or poor differentiation (*p*<0.001), pathological stage of III (*p*=0.006) or IV (p<0.001), T stage of T2 (*p*=0.005), T3 (p<0.001) or T4 (*p*<0.001), N stage of N1 (*p*<0.001), or N2 (*p*<0.001), and postoperative RT (*p*=0.001) had significantly worse DSS rates (Supplementary Table 1).

### The association of cleaved caspase-3 and caspase-3 expressions with tumorigenesis and clinicopathological outcomes in BMSCC patients

**Figure 1:**
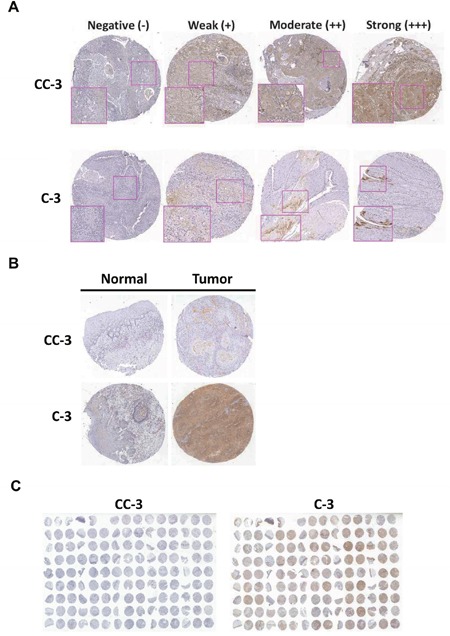
Immunoreactivity of cleaved caspase-3 (CC-3) and caspase-3 (C-3) in BMSCC **(A)** The representative photomicrographs for negative (–), weak (+), moderate (++) and strong (+++) staining of cleaved caspase-3 and caspase-3 in BMSCC tissues. **(B)** Comparative representative photomicrographs of cleaved caspase-3 and caspase-3 between normal and BMSCC tissues. **(C)** The representative photomicrographs of TMA sections for cleaved caspase-3 and caspase-3 staining.

To compare the expression levels of cleaved caspase-3 and caspase-3 between corresponding tumor-adjacent normal (CTAN) and tumor tissues, expression levels for these protein were determined using IHC. First, the intensity score of the cleaved caspase-3 and caspase-3 staining were measured using a numerical scale (0, no expression; 1, weak expression; 2, moderate expression; and 3, strong expression; Figure 1A). The protein levels of cleaved caspase-3 and caspase-3 were analyzed in BMSCC tissue cores and compared with the levels of the respective proteins in paired TAN tissue cores (Figure 1B) on the TMA (Figure 1C). After IHC scoring, expression levels of cleaved caspase-3 and caspase-3 were higher in tumor tissues (Table 1; CC-3 score: 2.93±2.03; C3 score: 4.55±1.44) compared to that in CTAN tissues (Table 1; CC-3 score: 0.02±0.19; C-3 score: 2.93±2.03). Moreover, fifty two out of 133 (39.1%) BMSCC patients showed ratio of less than 0 (CTAN/Tumor tissues) of cleaved caspase-3 expression since missing value ratio (because the cutoff values was 0 for cleaved caspase-3) were eighty out of 133 (60.2%) in BMSCC patients. In addition, the ratio of caspase-3 between CTAN and tumor tissues was decreased in nearly 70% BMSCC patients (Supplementary Table 2). These results indicated that the expression levels of both cleaved caspase-3 (Table 1; p<0.001) and caspase-3 (Table 1; p<0.001) in tumor tissues were significantly higher than those in CTAN tissues, suggesting these proteins might be associated with tumorigenesis. Moreover, the high expression of cleaved caspase-3 was correlated with advanced pathological stage (*p*=0.029) and larger tumor size (*p*=0.002) as shown in Table 2. However, the expression of caspase-3 was not associated with any pathological characteristics.

**Table 1:** The comparisons of cleaved caspase-3 (CC-3) and caspase-3 (C-3) expression in the tumor and tumor adjacent normal tissues of BMSCC patients

Variables	Tumor adjacent normal		Tumor		χ^2^	*p*-Value^*^
	Mean±SD	Median	Mean±SD	Median		
	(n=133)		(n=185)			
CC-3	0.02±0.19	0.00	0.85±1.20	0.00	72.116	**<0.001**
	(n=150)		(n=185)			
C-3	2.93±2.03	3.00	4.55±1.44	5.00	57.394	**<0.001**

SD, standard deviation.

^*^ *p* values were estimated by Kruskal-Wallis 1-way ANOVA test.

**Table 2:** Association of cleaved caspase-3 (CC-3) and caspase-3 (C-3) expression levels with demographical characteristics and pathological outcomes in BMSCC patients

Variable	No.	CC-3			C-3		
		Mean±SD	Median	*p-Value*	Mean±SD	Median	*p-Value*
Sex							
Female	4	0.50±1.00	0.00	0.557^*^	5.50±0.58	5.50	0.185^*^
Male	181	0.86±1.20	0.00		4.53±1.45	5.00	
Age, y							
≦40	25	0.44±0.82	0.00	0.338^†^	4.00±1.76	4.00	0.077^‡^
41-50	58	0.97±1.28	0.00		4.76±1.37	5.00	
51-60	56	0.91±1.21	0.00		4.75±1.25	5.00	
>60	46	0.85±1.23	0.00		4.35±1.51	5.00	
Cell differentiation							
Well	51	0.57±1.01	0.00	0.063^†^	4.20±1.51	5.00	0.069^†^
Moderate	125	0.99±1.27	0.00		4.74±1.33	5.00	
Poor	9	0.44±0.88	0.00		4.00±2.12	5.00	
AJCC pathological stage							
I, II	112	0.67±1.03	0.00	**0.029^§^**	4.55±1.42	5.00	0.979^*^
III, IV	73	1.12±1.37	0.00		4.55±1.49	5.00	
T classification							
T1, T2	140	0.70±1.12	0.00	**0.002^§^**	4.53±1.45	5.00	0.706^*^
T3, T4	45	1.31±1.33	1.00		4.62±1.45	5.00	
N classification							
N0	141	0.79±1.13	0.00	0.377^§^	4.50±1.43	5.00	0.356^*^
N1, N2	44	1.05±1.38	0.00		4.73±1.48	5.00	

AJCC, American Joint Committee on Cancer.

^*^p values were estimated by student's T test.

^†^*p values* were estimated by Kruskal-Wallis 1-way ANOVA test.

^‡^*p values* were estimated by 1-way ANOVA test.

^§^*p values* were estimated by Mann-Whitney U test.

### The association of cleaved caspase-3 and caspase-3 expressions with the survival of BMSCC patients

**Figure 2:**
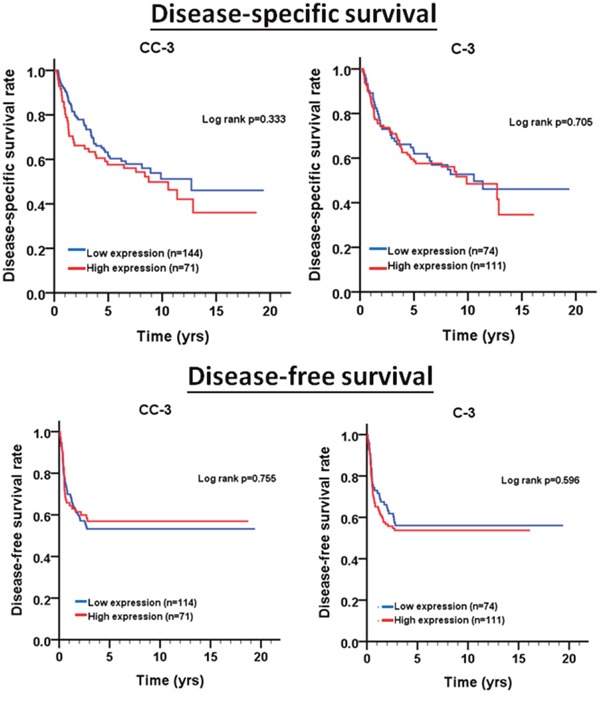
Survival curves depicting DSS and DFS of BMSCC patients according to the expression levels of cleaved caspase-3 (CC-3) and caspase-3 (C-3)

**Figure 3:**
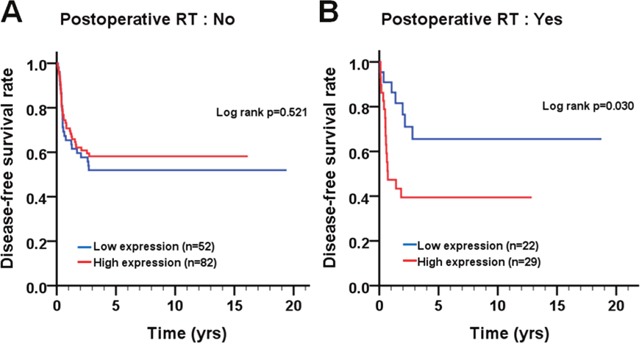
Survival curves depicting DFS of BMSCC patients according to caspase-3 expression pattern for patients **(A)** without and **(B)** with postoperative RT.

We further evaluated the relationship of cleaved caspase-3 and caspase-3 expressions with the survival of BMSCC patients. As results shown, cleaved caspase-3 and caspase-3 expressions were not associated with DSS and DFS in BMSCC patients regardless of univariate and multivariate analyses (Figure 2; Table 3). When the patients were stratified by postoperative RT, high expression of caspase-3 was associated with poor DFS (Figure 3, log-rank test *p*=0.030). In contrast, for patients without postoperative RT, caspase-3 expression was not significantly associated with DFS (*p*=0.521).

**Table 3:** The expression levels of cleaved caspase-3 (CC-3) and caspase-3 (C-3) for disease-specific survival and disease-free survival of BMSCC patients

Variable		No. (%)	Disease-specific survival				Disease-free survival			
			CHR	*p-Value*	AHR	p-Value^*^	CHR	*p-Value*	AHR	*p-Value*^†^
CC-3	Negative	114 (61.6)	1.00		1.00		1.00		1.00	
	Positive	71 (38.4)	1.23	0.334	0.87	0.547	0.93	0.755	0.81	0.374
C-3	Low	74 (40)	1.00		1.00		1.00		1.00	
	High	111 (60)	1.09	0.705	1.01	0.977	1.13	0.596	0.98	0.937
CC-3, C-3 expression										
	Negative, Low	45 (24.3)	1.00		1.00		1.00		1.00	
	Negative, High	69 (37.3)	1.66	0.069	1.40	0.239	1.40	0.220	1.25	0.433
	Positive, Low	29 (15.7)								
	Positive, High	42 (22.7)								

AJCC, American Joint Committee on Cancer; CHR, crude hazard ratio; CI, confidence interval; AHR, adjusted hazard ratio.

^*^p-value were adjusted for cell differentiation, AJCC pathological stage, postoperative RT by multiple Cox‘ s regression.

^†^*p-value* were adjusted for cell differentiation, AJCC pathological stage by multiple Cox‘ s regression.

### The association of the co-expression of cleaved caspase-3 and caspase-3 with the survival of BMSCC patients

**Figure 4:**
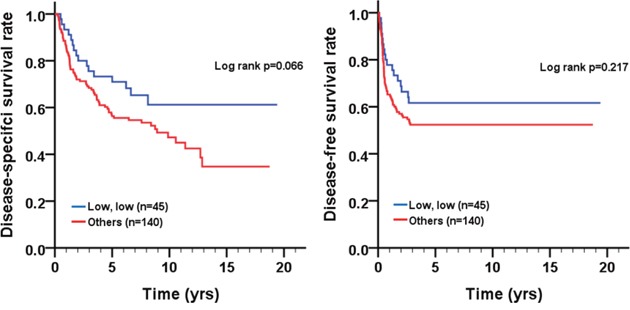
Survival curves of BMSCC patients according to the co-expression pattern of cleaved caspase-3 and caspase-3 **(A)** DSS and **(B)** DFS.

**Figure 5:**
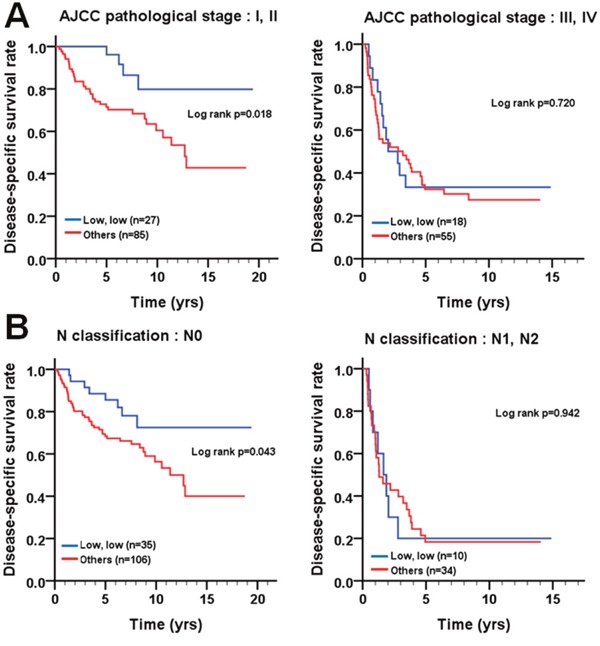
Survival curves depicting DSS of BMSCC patients according to the co-expression pattern of cleaved caspase-3 and caspase-3 stratified by **(A)** AJCC pathological stage (I + II and III + IV) and **(B)** lymph node invasion (N0 and N1 + N2).

We analyzed the association of the co-expression of cleaved caspase-3 and caspase-3 with the survival of patients before and after pathological stratification. Before pathological stratification, there was no difference in DSS ([cumulative hazard ratio, CHR]=1.66, 95% confidence interval (CI) 0.96-2.85, p=0.069; log-rank p=0.066) and DFS ([CHR]=1.40, 95% CI 0.82-2.38, *p*=0.220; log-rank *p*=0.217) in BMSCC patients with the low co-expression of cleaved caspase-3 and caspase-3 compared to those with the positive/high expression of either or both cleaved caspase-3 and caspase-3 (Table 3; Figure 4). However, patients with early pathological stage (I + II, p=0.018) or the absence of lymph node invasion (*p*=0.043) had a significantly longer DSS when they co-expressed low levels of cleaved caspase-3 and caspase-3 compared to the patients who expressed positive/high expression levels of either or both cleaved caspase-3 and caspase-3 (Figure 5). These results implied the possible role of the co-expression of cleaved caspase-3 and caspase-3 in BMSCC progression.

## DISCUSSION

Aberrant caspase-3 expression has been implicated in various types of cancer. For example, down-regulated cleaved caspase-3 expression has been correlated with gastric cancer [12], hepatocellular carcinoma [23] and prostate carcinoma [14]. However, elevated caspase-3 expression has been observed in tumor tissues of several cancer types, including glioblastoma [15], melanoma [16], acute myelogenous leukemia [24], breast carcinoma [17, 25], pancreatic ductal carcinoma [18], non-small cell lung cancer [19] and OSCC [21]. The possible reasons for aberrant caspase-3 expression may be that low expression of caspase-3 render cancer cells resistant to microenvironmental stresses [26, 27], and the overexpression of caspase-3 in dying cells may release growth-stimulating signals to allow non-apoptotic tumor cells to proliferate and survive under stressful conditions [22, 28]. To date, little is known about the role of caspase-3 expression in BMSCC. In this study, we evaluated the association of expression of cleaved caspase-3 and caspase-3 with tumorigenesis, pathological outcomes and prognosis using a TMA containing 185 BMSCC samples. Three major findings from IHC and statistical analyses are shown. First, the expression levels of cleaved caspase-3 and caspase-3 were higher in tumor tissues than those in tumor-adjacent normal tissues of BMSCC patients. Second, the high expression of cleaved caspase-3 was associated with advanced disease, such as poor pathological stage and large tumor size. Third, the high expression of caspase-3 and the positive/high expression of either or both cleaved caspase-3 and caspase-3 were associated with poor prognosis of BMSCC through stratification analysis.

Several studies have indicated that the caspase-3 expression is associated with many pathological outcomes (e.g., lymph node invasion, advanced clinical stage, and size of tumor) in various cancer types [29, 30]. In our study, the high expression of cleaved caspase-3 was associated with advanced stage and larger tumor size but not with cell differentiation or lymph node invasion in BMSCC. The association of high expression of cleaved caspase-3 with larger tumor size may imply the involvement of cleaved caspase-3 in proliferation of BMSCC [31, 32]. Moreover, the high expression of caspase-3 was also found to be associated with advanced stage in OSCC [21]. In contrast to our results, the high expression of caspase-3 was found to be associated with poor cell differentiation and lymph node invasion in OSCC [21], likely due to the fact that the association of caspase-3 expression with pathological outcomes in oral cancer is dependent on the site of cancer [33], as well as different environmental exposures and genetic alterations between Asian and Western BMSCC patients. These findings suggest that the involvement of caspase-3 in pathological outcomes is also varied among different types of cancer.

Previous studies have demonstrated that the high caspase-3 expression is significantly associated with poor prognosis in patients with gastric cancer, ovarian cancer, cervical cancer, colorectal cancer, non-small cell lung cancer, hepatocellular carcinoma, and breast cancer [34–38]. However, very few reports have shown the association of caspase-3 expression with prognosis of OSCC, especially for BMSCC. Our results indicated that higher caspase-3 expression was not significantly associated with DSS and DFS in BMSCC (Figure 2). However, through stratification analyses, the high expression of caspase-3 was found to be associated with recurrence in BMSCC patients with postoperative RT (Figure 3), which could serve as a remarkable biomarker for BMSCC patients. These findings also demonstrate the involvement of caspase-3 in radiation resistance for tumor recurrence [37]. Moreover, the positive/high expression of either or both cleaved caspase-3 and caspase-3 was associated with poor DFS in BMSCC patients with early pathological stage and without lymph node invasion (Figure 5), which could also provide a more accurate prognosis for BMSCC patients.

The potential mechanisms of caspase-3 in relation to poor DFS in BMSCC patient with postoperative RT could be that activated caspase-3 cleaves cytosolic calcium-independent phospholipase A2 (iPLA_2_) in dying tumor cells or tumor stromal cells in response to radiation [28, 39] to generate arachidonic acid, which is the precursor for the synthesis of prostaglandin E2 (PGE_2_) mediated by cyclooxygenase-1/-2 (COX1/2) [40]. Furthermore, inhibition of PGE_2_ synthesis or caspase-3 enhances the sensitivity of cancer cells to radiation therapy [28, 41]. Our results enable us to speculate that caspase-3 expression in tumor cells might activate iPLA_2_ and release PGE_2_ to promote radioresistance; however, this hypothesis needs to be further validated in the future. On the other hand, caspase cascades (caspase-3/8/9) are associated with cancer development in BMSCC patients (accepted). Correlating the expression of caspase-3 with other target molecules such as caspase-8 and caspase-9, might lead to the establishment of an effective prognostic immunohistochemical biomarker panel for BMSCC. Thus, caspase-3-specific inhibitors as well as antibodies, peptides, aptamers or siRNA directed against caspase-3 in combination with other anticancer agents could be used for the treatment of BMSCC patients, and more studies are required to further evaluate the therapeutic efficacy of these approached in the clinic.

One possible limitation of our study is the external validity of our findings limited to South Asia patients exposure to risk factors which are not specific to the Western population. To examine the validity, we used the other independent cohort from The Cancer Genome Altas (TCGA) database to validate the role of caspase-3 expression in prognosis of oral cancer patients from the Western population: (1) Consistent with our findings regarding the association of expression levels of the caspase-3 with “tumorigenesis” in BMSCC patients, the expression of caspase-3 (p=0.004, Supplementary Table 3) was significantly higher in oral cancer tissues than in tumor adjacent normal tissues, indicating that caspase-3 was very reliable biomarkers for tumorigenesis; (2) For “pathological outcomes” analysis, the high expression of caspase-3 was only significantly associated with age (p=0.006; Supplementary Table 4) in oral cancer patients but not with their pathological stage and tumor size; (3) For “survival” analysis, the high expression of caspase-3 was related to overall survival of oral cancer patients (p=0.02, Supplementary Table 5) Moreover, after stratification by clinicopathological outcomes, the high expression of caspase-3 was only related to poor overall survival of oral cancer patients with lymph node metastasis (Supplementary Figure 2) and larger tumor size (Supplementary Figure 3) but not of oral cancer patients with postoperative RT (Supplementary Figure 4). The possible reasons for the lack of consistency between our study and the results from the TCGA database may be that (a) the caspase-3 expression might be regulated by varied environmental and genotoxic exposures between Asian and Western oral cancer populations; (b) the prognostic role of caspase-3 regulated at the mRNA and protein levels may be varied in cancer [17, 25].

In conclusion, cleaved caspase-3 and caspase-3 could be biomarkers for tumorigenesis and prognosis, in particular, the high expression of caspase-3 is associated with the prognosis of BMSCC patients who receive postoperative RT, and the positive/high expression of either or both cleaved caspase-3 and caspase-3 is associated with prognosis of BMSCC patients with early pathological stage or without lymph node metastasis.

## MATERIALS AND METHODS

### Patients and tissues

A total of 185 BMSCC specimens as well as 150 CTAN tissues were obtained from Kaohsiung Veterans General Hospital between 1993 and 2006. The survival time was estimated from the time of surgery to 2013. Pathological TNM stage was determined at the time of initial resection of the tumor according to the guidelines of the 2002 American Joint Committee on Cancer system. All patients provided their informed consent for inclusion before they participated in the study. The study was conducted in accordance with the Declaration of Helsinki, and the protocol was approved by the Institutional Review Board (IRB) at the Kaohsiung Veterans General Hospital (IRB number: VGHKS96-CT1-08).

### Specimen characteristics and TMA construction

The constructed TMA block consisted of 149 cores, including 48 trios with each trio containing two cores from the tumor tissue and one core from the CTAN tissue of the same patient. Representative areas of BMSCC and CTAN were initially selected by an oral cancer pathologist (Dr. Ting-Ying Fu) from hematoxylin-eosin-stained sections for coring cylindrical tissues from paraffin-embedded tissues.

### Immunohistochemistry (IHC)

Rabbit monoclonal anti-caspase-3 antibody for the cleaved form (9661, Cell Signaling Technology, Beverly, MA) and rabbit monoclonal anti-caspase-3 antibody (sc-7148, Santa Cruz Biotechnology, Inc., Santa Cruz, CA) were utilized for IHC using a polymer-based system (Novocastra Novo-Link Max Polymer Detection System) according to the manufacturer's instructions [33].

TMA blocks were cut into 4-μm serial sections, deparaffinized in xylene, rehydrated in graded ethanol, and washed for 5 min with phosphate-buffered saline. Antigen retrieval was performed by immersion in Tris-EDTA (10 mM, pH 9.0) for 10 min at 125°C in a pressure cooker. Endogenous peroxidase activity was blocked with 3% hydrogen peroxide in methanol for 30 min. The slides were incubated overnight at 4°C in a humid chamber with anti-caspase-3 antibody for the cleaved form (dilution 1:100) and the anti-caspase-3 antibody (dilution 1:100) in Tris-buffered saline solution with 5% bovine serum albumin. Color was developed with a solution of 0.03% diaminobenzidine for 2 min at room temperature, and the sections were counterstained with hematoxylin.

### IHC analysis and scoring

Initially, an oral cancer pathologist (Dr. Ting-Ying Fu) accompanied by two pathology technicians evaluated the slides until all discrepancies were resolved. Subsequently, the two pathology technicians independently reviewed the slides except the cores with incorrect or uncertain contents, which were scored by the pathologist. During the evaluation, both of them were blinded to the clinical outcomes of the patients. The immunoreactivity was graded by a semiquantitative approach. Marker scores for cytoplasmic staining of cleaved caspasp-3 and caspase-3 were calculated based on staining intensity (-, negative; +, weak; ++, moderate; and +++, strong) and percentage of cells staining at each intensity level [0 (<5%), 1 (5-25%), 2 (26-50%), 3 (51-75%), and 4 (>75%)]. The intensity score and percentage of positive cells were then multiplied to produce the final scores (0-7). For survival analysis, the protein expression was dichotomized as negative/low expression (≤ the cutoff point) and positive/high expression (> the cutoff point) with the cutoff point set at the 25th percentile based on the distribution of the protein score. The cutoff values were 0 and 4 for cleaved caspase-3 and caspase-3 in BMSCC tissues, respectively. Moreover, the cutoff value of 0 was defined as negative expression, and the cutoff value of >0 was defined as positive expression for cleaved caspase-3. Based on the distribution of all cleaved caspase-3 (CC-3) and caspase-3 (C-3), setting the cutoff at 25% can yield relatively even distribution and enough patient number in each group (positive/high and negative/low expression groups; Supplementary Table 6).

### Statistical analysis

The SPSS software program (version 20.0, SPSS Inc., Chicago, IL, USA) was used for all statistical analyses. Student's t-test, Mann-Whitney U test, ANOVA and Kruskal-Wallis one-way ANOVA were used to evaluate the correlation between the expression of each protein and demographical characteristics and pathological outcomes. DSS was measured from the time of initial resection of the primary tumor to the date of cancer-specific death or last follow-up. DFS was calculated from the date of initial resection of the primary tumor to the date of recurrence or last follow-up. The cumulative survival curves were estimated using the Kaplan-Meier method. The comparison of the survival curves was performed by the log-rank test (for survival curve) and univariate Cox proportional hazards model (for CHR). A multivariate Cox proportional hazards model was used to determine independent predictors of survival using factors significant on the univariate analysis as covariates. A value of *p* < 0.05 was considered significant.

## SUPPLEMENTARY MATERIALS FIGURES AND TABLES



## Abbreviations

BMSCC: buccal mucosa squamous cell carcinoma
CHR: cumulative hazard ratio
CI: confidence interval
CT: chemotherapy
CTAN: corresponding tumor-adjacent normal
DFS: disease-free survival
DSS: disease-specific survival
IHC: immunohistochemistry
TMA: tissue microarray
RT: radiotherapy
