# Supplementary material for: Caspase-3 expression in tumorigenesis and prognosis of buccal mucosa squamous cell carcinoma
Source: Oncotarget. 2017 Aug 24;8(48):84237–47. doi: 10.18632/oncotarget.20494 (PMC5663591; doi:10.18632/oncotarget.20494)
Supplement: Supplementary file 1 [file oncotarget-08-84237-s001.pdf]

# Caspase-3 expression in tumorigenesis and prognosis of buccal mucosa squamous cell carcinoma

## SUPPLEMENTARY MATERIALS

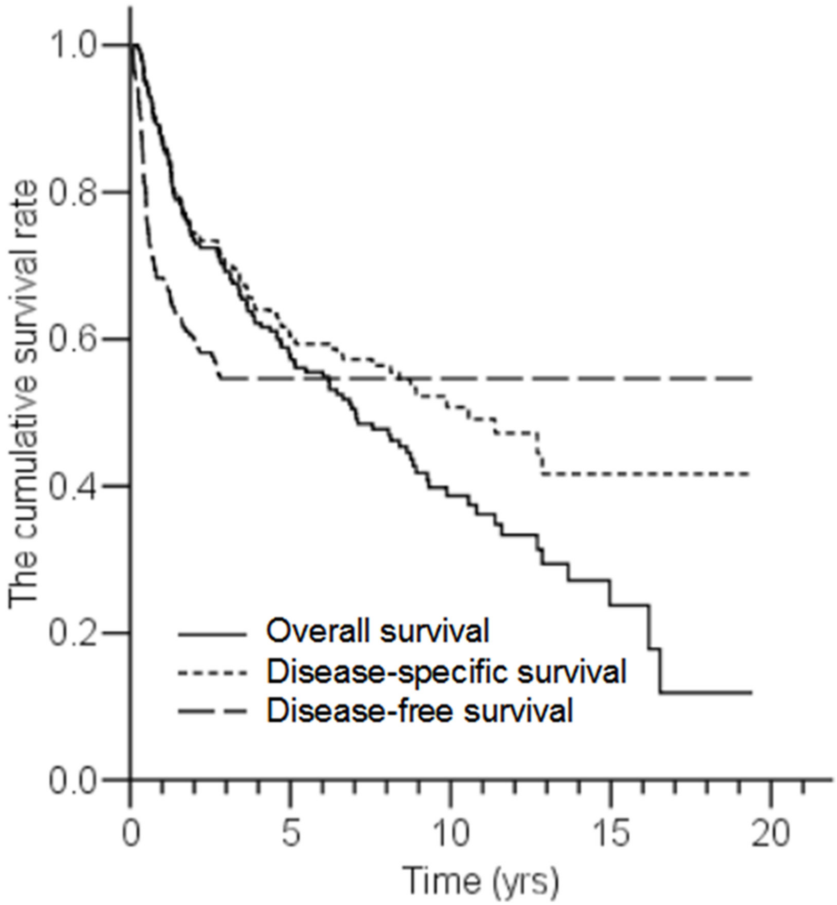


**Supplementary Figure 1: Cumulative survival rate for patients with BMSCC.**

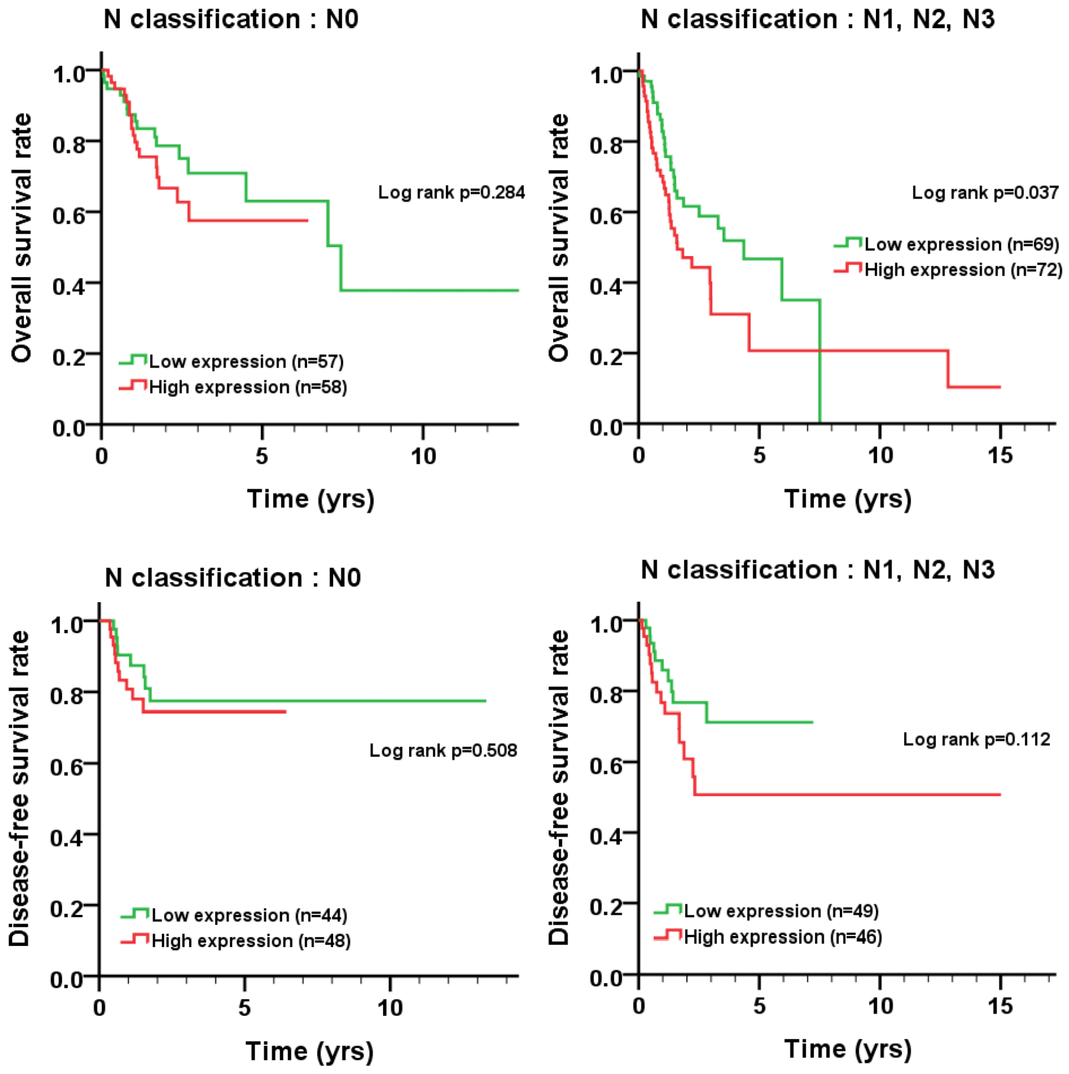


**Supplementary Figure 2: Survival curves depicting overall survival and disease-free survival according to caspase-3 expression pattern in oral cancer patients without (N0) and with (N+N2+N3) lymph node metastasis from TCGA database.**

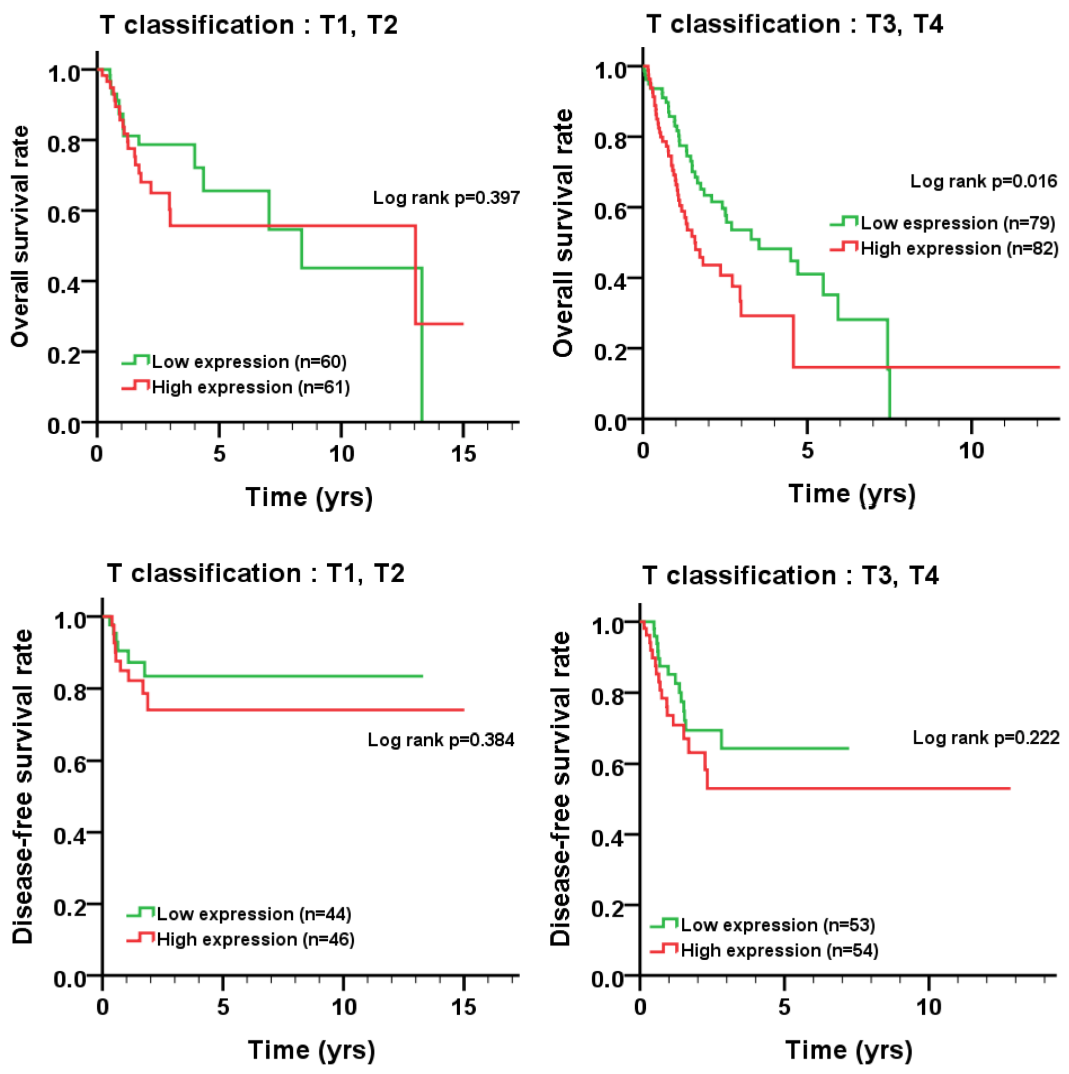


**Supplementary Figure 3: Survival curves depicting overall survival and disease-free survival according to caspase-3 expression pattern in oral cancer patients with small (T1+T2) and larger (T3+T4) tumor size from TCGA database.**

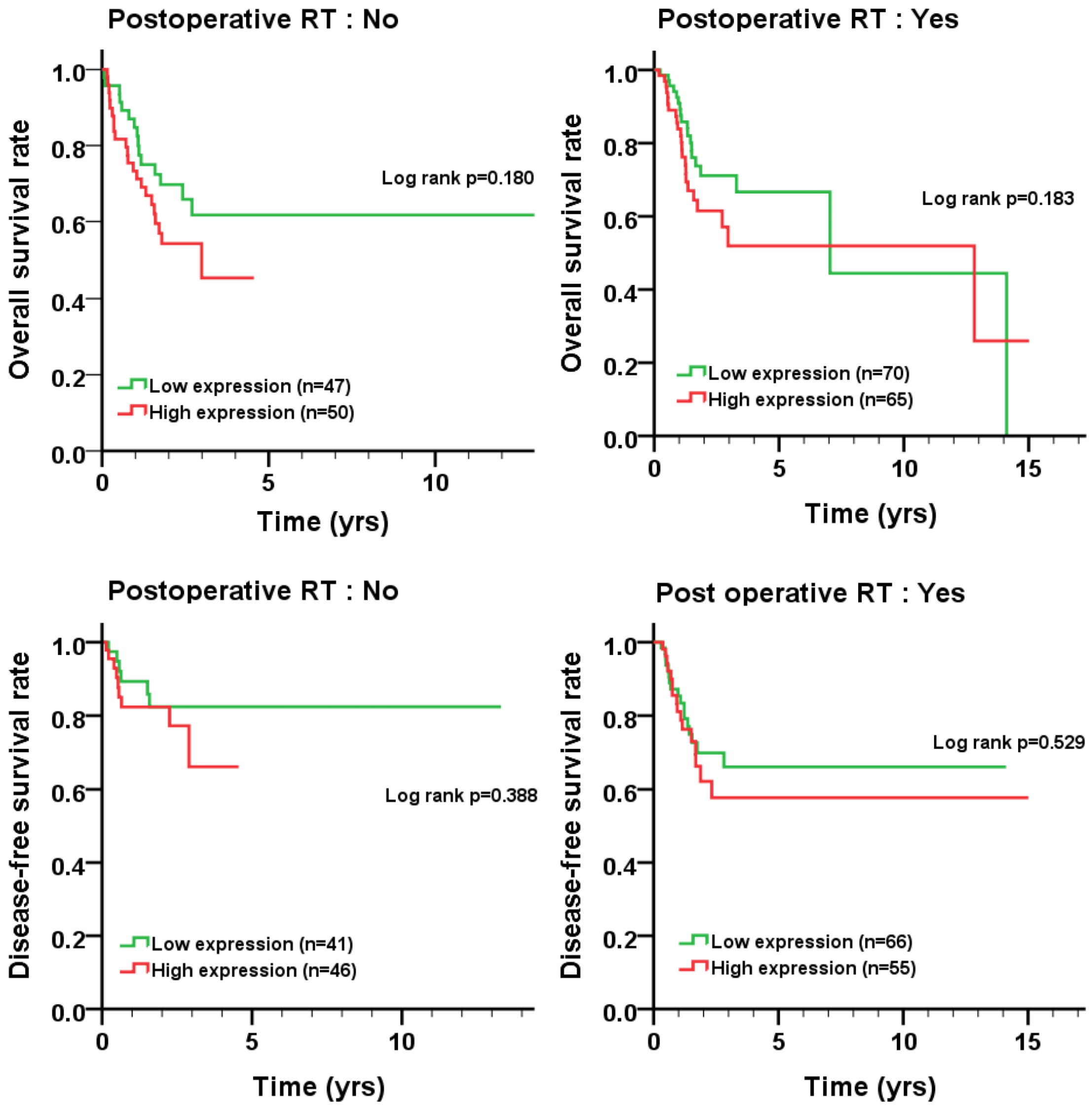


**Supplementary Figure 4: Survival curves depicting overall survival and disease-free survival according to caspase-3 expression pattern in oral cancer patients without (No) and with (Yes) postoperative RT from TCGA database.**

**Supplementary Table 1: Demographical charateristics and pathological outcomes and disease-specific survival of BMSCC patients**

| Variable | No. | CHR | *p-Value** |
|---|---|---|---|
| Sex | | | |
| Female | 4 | 1.00 | |
| Male | 181 | 0.49 | 0.227 |
| Age, y | | | |
| ≦40 | 25 | 1.00 | |
| 41-50 | 58 | 1.49 | 0.301 |
| 51-60 | 56 | 1.53 | 0.272 |
| >60 | 46 | 1.47 | 0.327 |
| Cell differentiation | | | |
| Well | 51 | 1.00 | |
| Moderate | 125 | **1.70** | **0.045** |
| Poor | 9 | **5.32** | **<0.001** |
| AJCC pathological stage | | | |
| I | 65 | 1.00 | |
| II | 47 | 1.30 | 0.428 |
| III | 19 | **2.81** | **0.006** |
| IV | 54 | **4.48** | **<0.001** |
| T classification | | | |
| T1 | 70 | 1.00 | |
| T2 | 70 | **2.14** | **0.005** |
| T3 | 12 | **4.58** | **<0.001** |
| T4 | 33 | **3.20** | **<0.001** |
| N classification | | | |
| N0 | 141 | 1.00 | |
| N1 | 19 | **2.90** | **<0.001** |
| N2 | 25 | **5.69** | **<0.001** |
| Postoperative RT | | | |
| No | 134 | 1.00 | |
| Yes | 51 | **2.31** | **<0.001** |
| Postoperative CT | | | |
| No | 181 | 1.00 | |
| Yes | 4 | 0.57 | 0.573 |

AJCC, American Joint Committee on Cancer; CHR, crude hazard ratio; RT, radiotherapy; CT, chemotherapy.
**p values* were estimated by Cox's regression.

**Supplementary Table 2: The score ratios of caspase-3 in CTAN/tumor tissues of BMSCC patients**

| Cleaved caspase-3 (CC-3) | | | |
|---|---|---|---|
| **CTAN/tumor ratio** | **Patient numbers** | **Percentage** | **Cumulative percentage** |
| 0.00 | 62 | 39.1 | 98.1 |
| 2.00 | 1 | 0.8 | |
| Total | 53 | 39.8 | |
| Missing value | 80 | 60.2 | |
| Total | 133 | 100.0 | |
| **Caspase-3 (C-3)** | | | |
| **CTAN/tumor ratio** | **Patient numbers** | **Percentage** | **Cumulative percentage** |
| 0.00 | 36 | 24.0 | 24.8 |
| 0.29 | 1 | 0.7 | 25.5 |
| 0.33 | 5 | 3.3 | 29.0 |
| 0.40 | 7 | 4.7 | 33.8 |
| 0.50 | 9 | 6.0 | 40.0 |
| 0.57 | 2 | 1.3 | 41.4 |
| 0.60 | 4 | 2.7 | 44.1 |
| 0.67 | 6 | 4.0 | 48.3 |
| 0.75 | 7 | 4.7 | 53.1 |
| 0.80 | 8 | 5.3 | 58.6 |
| 0.83 | 14 | 9.3 | 68.3 |
| 0.86 | 2 | 1.3 | 69.7 |
| 1.00 | 26 | 17.3 | |
| 1.20 | 5 | 3.3 | |
| 1.25 | 4 | 2.7 | |
| 1.33 | 1 | 0.7 | |
| 1.50 | 2 | 1.3 | |
| 1.67 | 2 | 1.36 | |
| 2.00 | 4 | 2.7 | |
| Total | 145 | 96.7 | |
| Missing value | 5 | 3.3 | |
| Total | 150 | 100.0 | |

**Supplementary Table 3: The comparison of caspase-3 expression between tumor adjacent normal and tumor tissues in oral cancer patients from TCGA database**

| Variables | Tumor adjacent normal | | Tumor | | Z | *p*-Value* |
|---|---|---|---|---|---|---|
| | Mean±SD | Median | Mean±SD | Median | | |
| | (n=29) | | (n=303) | | | |
| Caspase-3 | 66.44±8.65[d] | 67.36 | 71.19±6.87[cd] | 71.38 | -2.908 | **0.004** |

SD, standard deviation.
*p values* were estimated by Wilcoxon signed-rank test.
[a]p<0.001; [b]p<0.001; [c]p<0.001; [d]p<0.001; [e]p=0.011; [f]p=0.001.

**Supplementary Table 4: Association of caspase-3 expression with pathological outcomes in oral cancer patients from TCGA database**

| Variable | No. (%) | Caspase-3 | | |
|---|---|---|---|---|
| | | Mean±SD | Median | *p-Value* |
| Sex | | | | |
| Female | 100 (33.0) | 70.21±6.83 | 70.08 | 0.080* |
| Male | 203 (67.0) | 71.68±6.85 | 71.56 | |
| Age, y | | | | |
| ≦40 | 13 (4.3) | 66.70±6.13[abc] | 67.01 | **0.006‡** |
| 41-50 | 37 (12.2) | 71.76±5.96[a] | 71.21 | |
| 51-60 | 81 (26.7) | 71.58±6.58[b] | 71.65 | |
| 61-70 | 92 (30.4) | 69.85±7.25[d] | 70.14 | |
| >70 | 80 (26.4) | 72.82±6.76[cd] | 72.92 | |
| Cell differentiation | | | | |
| Well | 48 (16.0) | 69.59±6.65 | 69.60 | 0.173‡ |
| Moderate | 191 (63.7) | 71.36±6.56 | 71.49 | |
| Poor | 61 (20.3) | 71.95±7.65 | 72.65 | |
| AJCC pathological stage | | | | |
| I, II | 76 (27.1) | 71.41±6.33 | 71.21 | 0.772* |
| III, IV | 204 (72.9) | 71.15±6.73 | 71.41 | |
| T classification | | | | |
| T1, T2 | 123 (43.2) | 71.44±6.53 | 71.39 | 0.629* |
| T3, T4 | 162 (56.8) | 71.06±6.75 | 71.43 | |
| N classification | | | | |
| N0 | 117 (45.7) | 71.00±6.11 | 71.43 | 0.733* |
| N1, N2 | 139 (54.3) | 71.29±7.00 | 71.39 | |

AJCC, American Joint Committee on Cancer.
***p values* were estimated by student's T test.
†*p values* were estimated by Kruskal-Wallis 1-way ANOVA test.
‡*p values* were estimated by 1-way ANOVA test.
§*p values* were estimated by Mann-Whitney U test.
[a]p=0.022; [b]p=0.017; [c]p=0.003; [d]p=0.002.

**Supplementary Table 5: The expression levels of caspase-3 for overall survival and disease-free survival in oral cancer patients from TCGA database**

| Variable | | No. (%) | Overall survival | | | | Disease-free survival | | | |
|---|---|---|---|---|---|---|---|---|---|---|
| | | | CHR (95% CI) | *p-Value* | AHR (95% CI) | *p-Value** | CHR (95% CI) | *p-Value* | AHR (95% CI) | *p-Value*† |
| Caspase-3 expression | Low | 150 (50.0) | 1.00 | | 1.00 | | 1.00 | | 1.00 | |
| | High | 150 (50.0) | **1.52 (1.07–2.16)** | **0.020** | 1.45 (0.92–2.78) | 0.107 | 1.39 (0.80–2.43) | 0.246 | 1.25 (0.71–2.19) | 0.446 |

AJCC, American Joint Committee on Cancer; CHR, crude hazard ratio; CI, confidence interval; AHR, adjusted hazard ratio.
**p-value* were adjusted for cell differentiation(moderate, poor vs. well), AJCC pathological stage (stage II, III, IV vs stage I), postoperative RT by multiple Cox' s regression.
†*p-value* were adjusted for cell differentiation(moderate, poor vs. well), AJCC pathological stage (stage II, III, IV vs stage I) by multiple Cox' s regression.

**Supplementary Table 6: Distribution of scores for cleaved caspase-3 and caspase-3 expression in BMSCC patients**

| Cleaved caspase-3 (CC-3) | | | |
|---|---|---|---|
| **Score** | **Number** | **Percentage** | **Cumulative percentage** |
| 0.00 | 114 | 61.6 | 61.6 |
| 1.00 | 13 | 7.0 | 68.6 |
| 2.00 | 36 | 19.5 | 88.1 |
| 3.00 | 16 | 8.6 | 96.8 |
| 4.00 | 6 | 3.2 | 100.0 |
| Total | 185 | 100.0 | |
| **Caspse-3 (C-3)** | | | |
| **Score** | **Number** | **Percentage** | **Cumulative percentage** |
| 0.00 | 5 | 2.7 | 2.7 |
| 2.00 | 13 | 7.0 | 9.7 |
| 3.00 | 19 | 10.3 | 20.0 |
| 4.00 | 37 | 20.0 | 40.0 |
| 5.00 | 60 | 32.4 | 72.4 |
| 6.00 | 46 | 24.9 | 97.3 |
| 7.00 | 5 | 2.7 | 100.0 |
| Total | 185 | 100.0 | |
